# Linking folding dynamics and function of SAM/SAH riboswitches at the single molecule level

**DOI:** 10.1093/nar/gkad633

**Published:** 2023-07-31

**Authors:** Ting-Wei Liao, Lin Huang, Timothy J Wilson, Laura R Ganser, David M J Lilley, Taekjip Ha

**Affiliations:** Department of Biophysics, Johns Hopkins University, Baltimore, MD 21218, USA; Guangdong Provincial Key Laboratory of Malignant Tumor Epigenetics and Gene Regulation, Guangdong-Hong Kong Joint Laboratory for RNA Medicine, Sun Yat-Sen Memorial Hospital, Sun Yat-Sen University, Guangzhou 510120, China; Nucleic Acid Structure Research Group, MSI/WTB Complex, The University of Dundee, Dundee, Dow Street, Dundee DD1 5EH, UK; Department of Biophysics, Johns Hopkins University, Baltimore, MD 21218, USA; Nucleic Acid Structure Research Group, MSI/WTB Complex, The University of Dundee, Dundee, Dow Street, Dundee DD1 5EH, UK; Department of Biophysics, Johns Hopkins University, Baltimore, MD 21218, USA; Department of Biophysics and Biophysical Chemistry, Johns Hopkins University School of Medicine, Baltimore, MD 21205, USA; Howard Hughes Medical Institute, Baltimore, MD, USA

## Abstract

Riboswitches are regulatory elements found in bacterial mRNAs that control downstream gene expression through ligand-induced conformational changes. Here, we used single-molecule FRET to map the conformational landscape of the translational SAM/SAH riboswitch and probe how co-transcriptional ligand-induced conformational changes affect its translation regulation function. Riboswitch folding is highly heterogeneous, suggesting a rugged conformational landscape that allows for sampling of the ligand-bound conformation even in the absence of ligand. The addition of ligand shifts the landscape, favoring the ligand-bound conformation. Mutation studies identified a key structural element, the pseudoknot helix, that is crucial for determining ligand-free conformations and their ligand responsiveness. We also investigated ribosomal binding site accessibility under two scenarios: pre-folding and co-transcriptional folding. The regulatory function of the SAM/SAH riboswitch involves kinetically favoring ligand binding, but co-transcriptional folding reduces this preference with a less compact initial conformation that exposes the Shine–Dalgarno sequence and takes min to redistribute to more compact conformations of the pre-folded riboswitch. Such slow equilibration decreases the effective ligand affinity. Overall, our study provides a deeper understanding of the complex folding process and how the riboswitch adapts its folding pattern in response to ligand, modulates ribosome accessibility and the role of co-transcriptional folding in these processes.

## Graphical Abstract

**Graphical Abstract:**
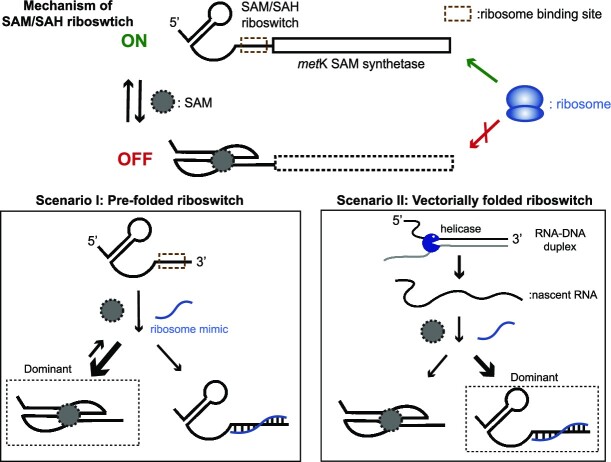

## INTRODUCTION

Riboswitches are regulatory units of RNA that mediate gene expression in response to binding of specific metabolites. They are widely found in bacteria (1–3) but also exist in archaea (4), plants (5) and fungi (6,7). To date, >40 classes of riboswitches have been discovered, and they bind chemically diverse ligands and contribute up to 4% to the bacteria genetic control, especially in gram positive bacteria. Riboswitches are mostly located at the 5′-untranslated regions (5′-UTR), upstream of the regulated genes, and include an aptamer domain capable of binding a particular metabolite with exceptionally high specificity. The riboswitch adopts a specific fold on binding the ligand, leading to up- or down-regulation of the gene either by altering transcription or translation. Since the riboswitch folds and acts as a regulatory unit during transcription, the timing of ligand binding and conformational change is critical, necessitating investigation into its folding kinetics. Substantial research has been devoted to the origins of specificity, correlation of sequence and structure (8,9), folding kinetics (10–12), and identification of candidates that can be adapted for drug-delivery (13) and *in vivo* imaging (14).

S-adenosylmethionine (SAM)-binding riboswitches comprise one of the largest classes of riboswitches (1). SAM is synthesized from methionine and ATP by SAM synthetase, encoded by the *metK* gene. SAM is an essential co-substrate of methyltransferases, supplying the methyl group for methyl transfer. Once the methyl group is donated, the resulting *S*-adenosyl-l-homocysteine (SAH) is degraded due to its toxicity (15,16). To maintain SAM concentration, SAM acts as an inhibitor of MetK synthesis (17–20). This regulation is achieved by the SAM-riboswitches, which bind SAM and acts as negative feedback unit for genes in methionine or cysteine biosynthesis. When SAM concentration goes up, expression of genes in methionine or cysteine biosynthesis is reduced by its upstream 5′-UTR adopting translation-off conformation.

Six sub-classes of SAM riboswitches (SAM-I to SAM-VI) have been identified, classified into three families according to their structural features (17–25). In general, SAM riboswitches exhibit strong discrimination between SAM and SAH by electrostatically interacting with the positive-charged sulfonium cation of the SAM molecule (26–33), as previously shown by X-ray crystallography and single-molecule methods (22,34). By contrast, the SAM/SAH riboswitch does not discriminate between SAM and SAH (35,36).

The ligand binding interactions of this particular SAM/SAH riboswitch have been previously revealed by NMR and X-ray crystallography (35,36). Binding of SAM or SAH is accompanied by the formation of three base pairs that extend the helix of the stem-loop P1, and formation of a pseudoknot helix PK (Supplementary Figure S1A). The ligand binds in the major groove of the extended helix, with the methionyl nitrogen and the adenine moiety hydrogen bonded to a specific cytosine nucleobase. The methionine side chain containing the sulfonium of SAM or the thioether of SAH does not make any direct contact with the RNA, which explains the inability to distinguish between the two ligands. Single-molecule FRET (fluorescence resonance energy transfer) (37) was utilized to compare the binding of SAM and SAH and their kinetic characteristics (36), and no significant differences were observed between the two ligands. Although the ligand-bound state and basic kinetics have been characterized, important features such as ligand-free conformations, binding, folding kinetics, and its role in modulating translation initiation activity are still unknown.

Here, we used single-molecule FRET to investigate the ligand-free and ligand-bound conformations of the SAM/SAH riboswitch and map the energy landscape of folding dynamics and altered ribosome accessibility. Folding of the riboswitch is highly heterogeneous, suggesting a rugged conformational landscape that allows for sampling of the ligand-bound conformation even in the absence of ligand. The addition of ligand shifts the landscape, favoring the ligand-bound conformation. Site specific mutations showed that the PK helix is crucial for determining ligand-free conformations and their ligand responsiveness. In addition, we investigated the accessibility of the ribosomal binding site under two scenarios: (i) pre-folding of the riboswitch: folding equilibrium is reached in advance and (ii) vectorial release of the RNA by mimicking co-transcriptional folding. Vectorial folding initially favors an open conformation that exposes the ribosome binding site, and it takes min before conformational redistribution to that of the pre-folded riboswitch. Such slow equilibration decreases the effective ligand affinity. Overall, our studies offer a deeper understanding of the complexity of the folding process, revealing the mechanism by which the riboswitch adapts its folding pattern in response to ligand and modulates ribosome accessibility, and how co-transcriptional folding influences these processes.

## MATERIALS AND METHODS

Riboswitch ligands SAM (A7007), SAH (A9384) were all obtained from Sigma. The RNAs (wide-typed and mutants) are synthesized as described in the following sections. DNA oligonucleotides for mimicking ribosome binding were purchased from Integrated DNA Technologies (Coralville).

### RNA synthesis for single-molecule experiments

The wild-type and mutated SAM/SAH riboswitches for single molecule measurements contain a Cy3 fluorophore attached to the O2’ of U20 generated by Cu^2+^-catalyzed reaction of alkyne-modified RNA with an azide-attached fluorophore (Lumiprobe Corp). The wild-typed RNA had an 18 nt 3′ DNA extension for base-pairing to the anchor DNA, and the complete sequence was (DNA starts with d and underscored): GAUACCUGUCACAACGGCU(U-Cy3)CCUGGCGUGACGAGGUGACCUCAGUGGAGCAA d(ACCGCTGCCGTCGCTCCG), and all the other mutated sequences were showed in Supplementary Table S1.

The anchor DNA had a 5′-biotin and 3′ Cy5 fluorophore and was complementary to the 18-nt extension of the SAMSAH riboswitch strand. Its sequence was: biotin-CGGAGCGACGGCAGCGGT-Cy5. RNA oligonucleotides were synthesized using t-BDMS phosphoramidite chemistry (38) as described in Wilson et al. (39), implemented on an Applied Biosystems 394 DNA/RNA synthesizer. RNA was synthesized using ribonucleotide phosphoramidites with 2 -Otter-butyldimethyl-silyl (t-BDMS) protection (Link Technologies) (40,41). Oligonucleotides containing 5-bromocytidine (ChemGenes) were deprotected in a 25% ethanol/ammonia solution for 36 h at 20°C. All oligoribonucleotides were re-dissolved in 100 μl of anhydrous DMSO and 125 μl triethylamine trihydrofluoride (Sigma-Aldrich) to remove t-BDMS groups, and agitated at 65°C in the dark for 2.5 h. After cooling on ice for 10 min, the RNA was precipitated with 1 ml of butanol, washed once with 70% ethanol and suspended in double-distilled water. RNA was further purified by gel electrophoresis in polyacrylamide under denaturing conditions in the presence of 7 M urea. The full-length RNA product was visualized by UV shadowing. The band was excised and electroeluted using an Elutrap Electroelution System (GE Healthcare) into 45 mM Tris-borate (pH 8.5), 5 mM EDTA buffer for 12 h. at 150 V at 4°C. The RNA was precipitated with isopropanol, washed once with 70% ethanol and suspended in water or ITC buffer (40 mM HEPES-K (pH 7.0), 100 mM KCl, 10 mM MgCl_2_).

### Ligand titration of pre-folded riboswitches: wild-typed and mutated riboswitches

40 pM of the pre-annealed SAM/SAH riboswitch molecules were immobilized on a neutravidin-functionalized, polymer-passivated surface and free molecules were washed out with T50 buffer. Image buffer containing an oxygen-scavenging system was freshly mixed before measurements, comprising 1% (w/v) dextrose, 2 mM Trolox, glucose oxidase (1 mg/ml; Sigma-Aldrich), and catalase (500 U/ml; Sigma-Aldrich)] in buffer containing 40 mM HEPES (pH 7.5), 100 mM KCl, 2 mM MgCl_2_. All the ligands were diluted with image buffer immediately prior to measurements. The ligands were incubated for 5 min before imaging. Short movies (duration of 1.5 s: 20 frames) were collected for 30 field of view for generating distribution of FRET efficiencies (*E*_FRET_). The distribution is then fitted by two individual Gaussian function, and the high *E*_FRET_ ratio is estimated accordingly.

### Single-molecule imaging and data acquisition

Single-molecule FRET data were obtained using a prism-based total internal reflection fluorescence (TIRF) microscope. The Cy3 and Cy5 fluorophores were excited by a 532-nm laser (Coherent Compass 315M) and a 638-nm laser (Cobolt 06-MLD) respectively. The fluorescence emission was collected by a water immersion objective (Olympus NA 1.2, 60×) and recorded by a back-illuminated electron-multiplying charge-coupled device camera (iXON, Andor Technology) with a dual-view setup. The dual-view setup used a long-pass emission filter (Semrock BLP02-561R-25) for eliminating the 532-nm laser, and a notch filter (Chroma ZET633TopNotch) for eliminating the 638-nm laser. The fluorescence emission was separated into donor and acceptor emission by a long-pass dichroic mirror (Semrock FF640-FDi01-25X36). The passivated PEG quartz slides and coverslips were purchased from Johns Hopkins Slides Core and were assembled into a reaction chamber. (42) Spots detection, background subtraction, donor leakage and acceptor direct-excitation correction followed our previous protocol (42). Custom codes are available on GitHub (https://github.com/Ha-SingleMoleculeLab) and archived in Zenodo with the following doi. Data acquisition DOI: 10.5281/zenodo.4925630; Raw data analysis DOI: 10.5281/zenodo.4925617.

### Single-molecule data analysis of pre-folded riboswitch

Single-molecule traces showing *E*_FRET_ as a function of time were categorized into three types of behavior. (i) *E*_FRET_ remained middle for the duration of observation, up to 1 min, (ii) *E*_FRET_ remained high for the duration of observation (iii) *E*_FRET_ fluctuated between the middle and high values. To characterize further the dynamic species, the regions of dynamics were collected and analyzed by ebFRET (43) and the two-state dwell time was plotted into log-scale scatter plot.

### Single-molecule data analysis of vectorially folded riboswitch

Single-molecule traces showing the immobilized heteroduplex was unwound were categorized into four types of behavior. We classified the riboswitch folding behavior into four types (Supplementary Figure S7A): (i) molecules transitioned from the heteroduplex state to the closed conformation without any detectable intermediate, then remaining there, (ii) molecules transitioned from the heteroduplex state to the open conformation, then remaining there, (iii) molecules transitioned from the heteroduplex state to one undergoing fluctuations between the open and closed conformations, (iv) molecules transitioned from the heteroduplex to fluctuating states after which they became locked in the closed conformation.

### Pseudo-functional readout of ribosome accessibility of pre-folded riboswitch

40 pM of the pre-annealed SAM/SAH riboswitch molecules were immobilized on a neutravidin-functionalized, polymer-passivated surface and free molecules were washed out with T50 buffer. All the ligand (SAM) and DNA oligonucleotides with a designated concentration was freshly mixed with image buffer containing an oxygen-scavenging system. Short movies (duration of 1.5 sec: 20 frames) were collected for 30 field of view immediately or at designated time (5-min or 1 h) after injection for generating distribution of FRET efficiencies (*E*_FRET_).

### Sample preparation for the single-molecule FRET measurements

For preparation of the pre-folded assay, 10 μM of the SAM/SAH riboswitch molecule or the riboswitch mutants with internal Cy3 labeled was annealed with 15 μM anchored DNA with Cy5 and biotin label under 1× T50 [10 mM Tris (pH 8.0), 50 mM NaCl) buffer followed by slow cooling from 95°C to room temperature.

For preparing of the vectorial folding assays, 10 μM anchored DNA with Cy5 and biotin label was annealed with 20 μM of the Cy3-labeled SAM/SAH riboswitch and 40 μM complementary DNA oligos (cDNA) with dT30 overhang in 10 μl of 1× T50 [10 mM Tris (pH 8.0), 50 mM NaCl] by incubating the mixture at 95°C for 1 min, 75°C for 5 min, 37°C for 15 min and finally equilibrating at room temperature for 5 min (44,45).

### Vectorial folding as a mimic of riboswitch folding and ligand binding

Labeled and biotinylated heteroduplexes were immobilized on a neutravidin-functionalized surface and free heteroduplexes were washed out. 50 nM of Rep-X (46) was incubated for 2 min with heteroduplexes in the imaging buffer (40 mM HEPES (pH 7.5), 100 mM KCl, 2 mM MgCl_2_) containing an oxygen-scavenging system, and images (duration of 1.5 s: 20 frames) were collected for 30 field of view for confirming the heteroduplex conformation. Unwinding was initiated by mixing unwinding buffer with/without ligands at designated concentration. Unless specified otherwise, the unwinding buffer contained 40 mM HEPES (pH 7.5), 100 mM KCl, 2 mM MgCl_2_, 2 mM ATP with an oxygen-scavenging system. Buffer with ATP then triggered the pre-bound RepX into unwinding the anchored heteroduplex.

For the real-time observation (‘flow-in’ experiments) of riboswitch released from heteroduplex, imaging was started 12 s before the addition of the unwinding buffer. For characterization of VFA products after helicase unwinding, images were taken after the addition of the unwinding buffer at designated time. The loading and unwinding buffers used during imaging contained additional 1% (w/v) dextrose, 2 mM Trolox, glucose oxidase (1 mg/ml; Sigma-Aldrich), and catalase (500 U/ml; Sigma-Aldrich).

### Vectorial folding with ligand and ribosome mimic addition simultaneously

Labeled and biotinylated heteroduplexes were incubated with 50 nM Rep-X as previously described, images were taken before addition to confirm the heteroduplex conformations. Ligands, ribosome mimics (oligonucleotides with 9-nt or 15-nt complementary to ribosome binding site), and additional Rep-X (50 nM for 9-nt; 100 nM for 15-nt) were mixed with unwinding buffer. The additional Rep-X is added in order to reduce the competition of free oligonucleotides to the Rep-X pre-incubated before unwinding mixture. This optimized condition is tested with a negative control, where additional Rep-X with dT9 or dT15 were added simultaneously into the heteroduplex, no observable loss of unwinding efficiency in this condition. The negative control experiments were shown in Supplementary Figure S10.

## RESULTS

### Heterogeneous folding energy landscape of ligand-free riboswitch

**Figure 1.**
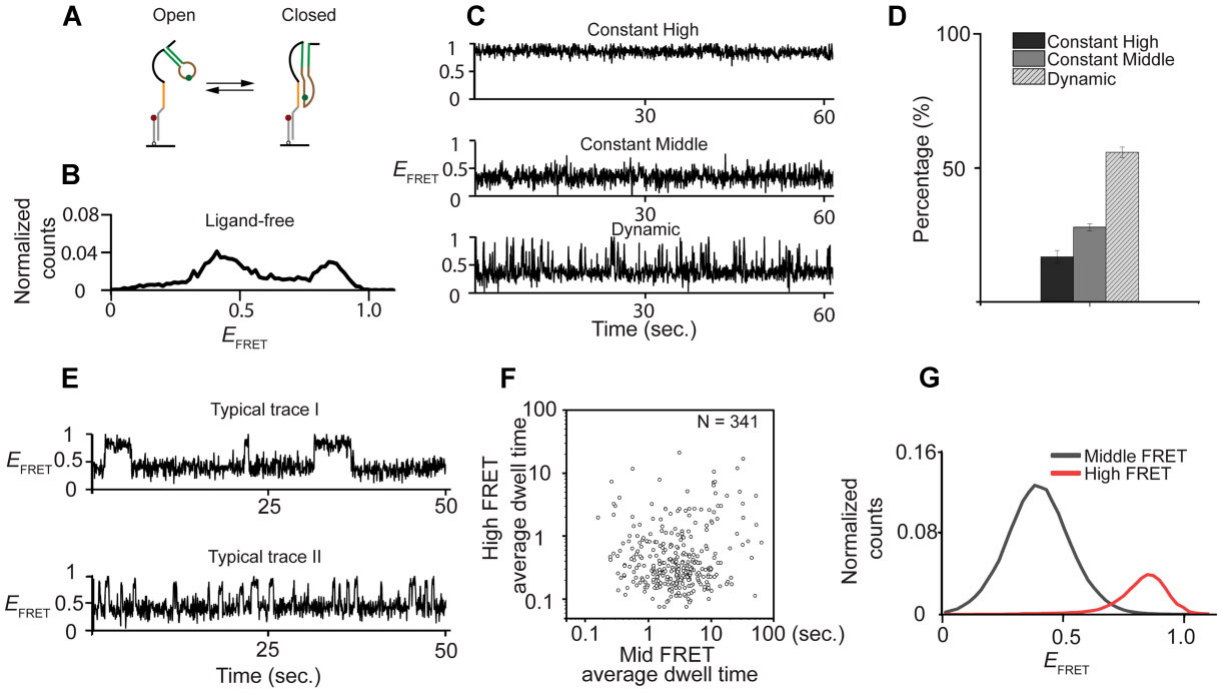
Studies of ligand-free conformations of the SAM/SAH riboswitch by single-molecule FRET. (**A**) A scheme showing the probable folding of SAM/SAH riboswitch RNA. An 18 nt DNA molecule with a 3′ Cy5 acceptor (red circle) was attached via its biotinylated 5′ terminus to a quartz slide. Cy3 donor (green circle) was attached to the bulged nucleotide in the PK helix of the riboswitch, and an 18 nt 3′ DNA extension complementary to the surface-attached DNA allowed the riboswitch to be tethered to the slide. If the pseudoknot helix is not formed the fluorophores should be separated (the open conformation with mid FRET efficiency) whereas in the folded structure the fluorophores should be much closer (the closed conformation with high FRET efficiency). (**B**) Distribution of FRET efficiencies (*E*_FRET_) for SAM/SAH riboswitch molecules corresponding to the open and closed conformations. (**C**) Characteristic traces of *E*_FRET_ as a function of time recorded. Three representative traces are shown, illustrative of constant high FRET (top), constant mid FRET (middle) and dynamic molecules (bottom) undergoing transitions between states of high and middle *E*_FRET_. (**D**) Histograms showing the relative fraction of constant high FRET (black), constant middle FRET (dark gray) and dynamic molecules (light grey with line). (**E**) Among traces showing dynamic transitioning, two characteristic traces are shown, indicating the kinetics of transitioning is diverse and heterogenous. (F, G) Such diverse transitioning kinetics is then fitted into two states transitioning by ebFRET. Individual molecule average dwell time is plotted into log-scale scatter plot (**F**), indicating transitioning heterogeneity. And the high FRET and middle FRET probability within the dynamic population is plotted individually (**G**).

First, we determined the conformational dynamics of the SAM/SAH riboswitch in the absence of ligand. Single riboswitch molecules were tethered to the quartz slide by hybridization of a 3′ extension to an oligonucleotide carrying a Cy5 acceptor at its 3′ terminus. The riboswitch construct has a Cy3 donor attached internally within the loop region such that FRET efficiency, *E*_FRET_, between the two fluorophores can be used to distinguish between conformations. We anticipated two major conformations: the open state with a stem-loop structure previously determined by in-line probing and the closed state with a H-type pseudoknot (Figure 1A, (31)). The closed conformation likely has a global conformation similar to the crystal structure of the liganded riboswitch that showed the 8-bp extended P1 helix and 5-bp pseudoknot (PK) helix coaxially stacked with each other (36). We previously showed that Cy3 labeling in the loop region does not perturb folding (36).

Single-molecule histograms of *E*_FRET_ showed two major peaks, likely corresponding to the open conformation (mid- *E*_FRET_ = 0.4, Figure 1B) and the closed conformation (high- *E*_FRET_ = 0.84, Figure 1B), suggesting the ligand-bound conformation is adopted even without ligand. Lowering magnesium concentration reduced the high- *E*_FRET_ population but a significant percentage (>30%) of high- *E*_FRET_ population remained even in the absence of Mg^2+^ (Supplementary Figure S1B), suggesting divalent cations promote the closed conformation, but are not required.

Single-molecule time traces of *E*_FRET_ displayed three types of behavior: (i) constant mid-FRET (*E*_FRET_ = 0.4) (ii) constant high-FRET (*E*_FRET_ = 0.84) and (iii) dynamic behavior showing transitions between mid- and high-FRET values (Figure 1C). The majority (55%, Figure 1D) of traces showed dynamic behavior, further indicating that even in the ligand-free state the closed conformation is sampled. The interconversion kinetics of the dynamic population was quantified by calculating the average dwell times for high and mid-*E*_FRET_ states for each molecule and visualized as a log-scale scatter plot. The average dwell times covered a wide range, spanning up to 3 orders of magnitude (Figure 1E and F). In most cases, the open conformation was longer-lived than the closed conformation (Figure 1G). The dynamic transitioning was a long-lasting characteristic with no clear population interconversions to or from constant mid- or high-FRET states within our experimental window, up to 50 min long (a typical trace shown in Supplementary Figure S2A with zoom-in traces in Supplementary Figure S2B-D, with intermittent 30 s exposure every 5 min). We attribute this ‘static heterogeneity’ to deep energy wells, and our preliminary investigation at higher temperature still showed static heterogeneity.

### Ligand binding reshapes the folding energy landscape

**Figure 2.**
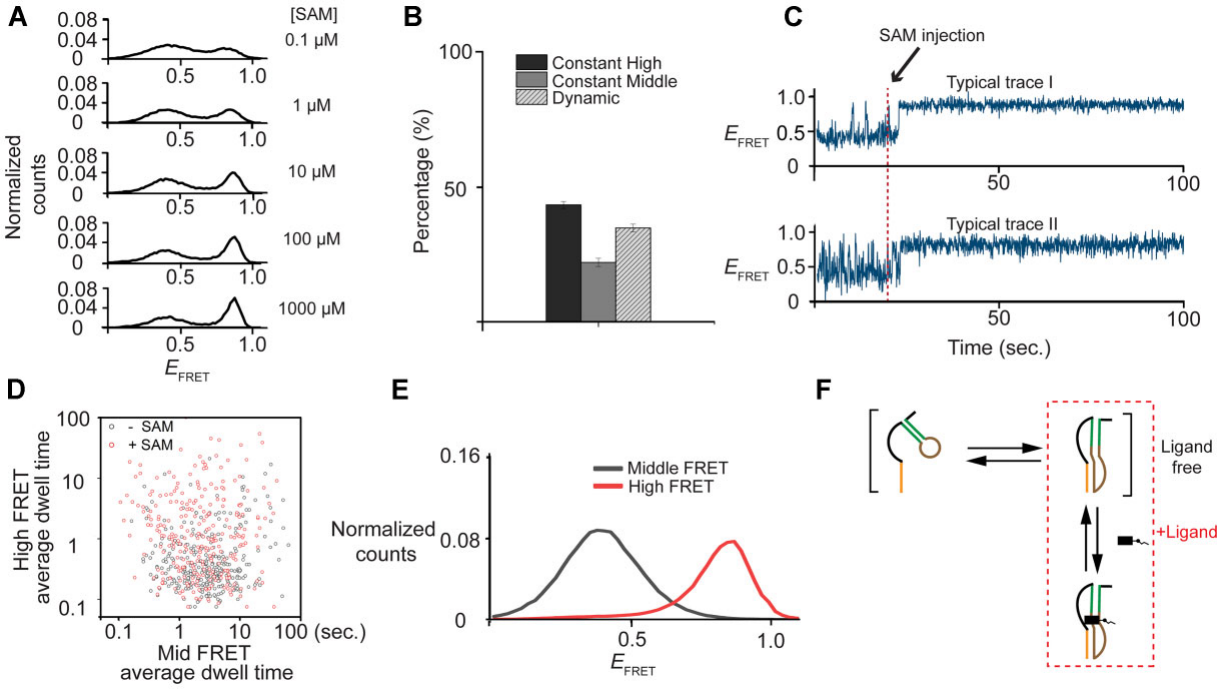
Studies of ligand-induced conformations of the SAM/SAH riboswitch by single-molecule FRET. (**A**) Distribution of FRET efficiencies (*E*_FRET_) as a function of SAM and (**B**) the histograms showing the relative fraction of constant high FRET (black), constant middle FRET (dark gray) and dynamic molecules (light grey with line) in the presence of 1 mM SAM. (**C**) Two typical trajectories of riboswitches showed populations converted from dynamics to constant high FRET while SAM was flowed into the reaction chamber at 20 s, corresponding to the change of the relative populations in the presence of SAM. (**D, E**) Among molecules remained transitioning, transitioning kinetics was then fitted into two states by ebFRET. Individual molecule average dwell time is plotted into log-scale scatter plot (**D**). And the high FRET and middle FRET probability within the dynamic population is plotted individually (**E**). (**F**) A scheme showing in the presence of ligand, conformations are shifted toward the closed conformations.

Next, we measured riboswitch folding in the presence of the cognate ligand SAM. The high-FRET state indeed represents the closed conformation because SAM increased the high-FRET population (Figure 2A). SAM concentrations we used in our study are similar to the physiological concentration in E. coli, ranging from 28 μM to 228 μM (20). The fraction of molecules in the high-FRET population vs ligand concentration could be fitted using a simple two-state binding isotherm, yielding a dissociation constant (*K*_d_) of 10 μM, similar to those measured in bulk solution using isothermal calorimetry (36). The fraction of molecules in the constant high-FRET species increased from 0.16 to 0.43, and this increase appeared to occur at the expense of the dynamic species while the population of the constant mid-FRET species remained unchanged upon ligand binding (Figure 2B). This suggests that the molecules already in dynamic exchange with the closed conformation were more readily locked into the closed conformation via ligand binding, while the constant mid-FRET population may be trapped in a misfolded state that is not easily rescued by ligand binding. Indeed, flow experiments demonstrated that ∼43% of dynamic species (37 of 86) showed clear locking into the closed conformation after addition of 1 mM SAM (Figure 2C). Notably, a significant fraction (38%) of molecules still exhibited dynamic transitioning even when excess ligand was added. However, the average high-FRET dwell time became significantly longer upon ligand addition (Figure 2D). A schematic model is presented in Figure 2F, which illustrates that in the absence of ligand, the riboswitch is in a dynamic equilibrium between folded and unfolded states, and that the addition of a ligand results in a shift towards the closed conformation.

### Structural perturbations provide insights into the folding energy landscape

**Figure 3.**
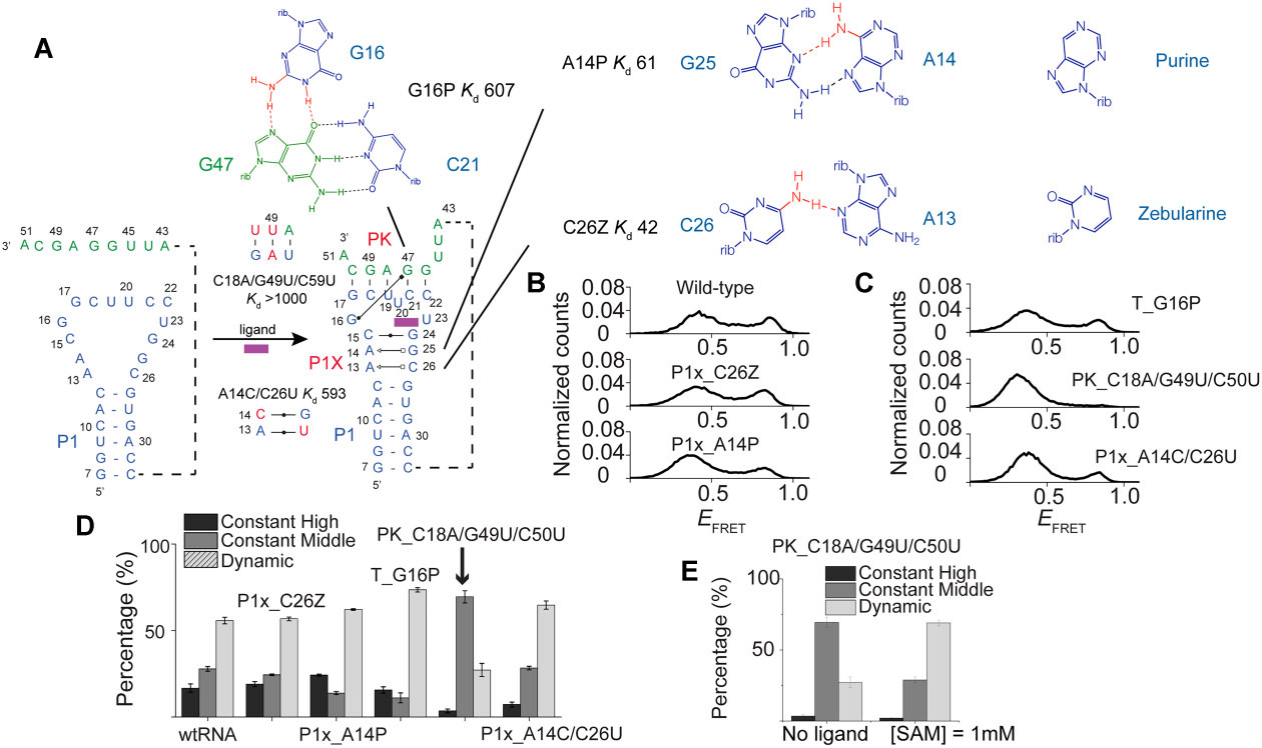
Studies of mutations at local structures affect conformations and ligand responsiveness. (**A**) The ligand-bound riboswitch previously revealed by X-ray crystallography (36) adopts H-type pseudoknot structure with three stabilizing features: (i) P1x: the extension to helix P1, comprising one W-C base pair and two non-W-C pairs, (ii) PK: the pseudoknot helix, involving the Shine–Dalgarno sequence and (iii) a triple base interaction (G47:C16-G16). (B, C) Distribution of FRET efficiencies (*E*_FRET_) of the ligand-free conformations of, (**B**) wild-type, P1x_C26Z and P1x_A14P mutants; (**C**) T_G16P, PK_C18A/G49U/C50U and P1x_A14C/C26U mutants. All mutations shared similar folding behaviors of constant high FRET, constant middle FRET, and dynamics. (**D**, **E**) The histograms showing the relative fraction of constant high FRET (black), constant middle FRET (dark gray) and dynamic molecules (light grey) of wild-type and all mutants (D). Among all mutants, PK_C18A/G49U/C50U showed the most different populations both in the absence or in the presence of ligands (E).

We next examined alterations in the conformational landscape caused by mutations designed to impact the local structural stability. As shown in Figure 1A and Figure 3A, the ligand-bound riboswitch adopts H-type pseudoknot structure with three stabilizing features: (i) P1x: the extension to helix P1, comprising one W-C base pair and two non-W–C pairs, (ii) PK: the pseudoknot helix, involving the Shine–Dalgarno sequence and (iii) T: a triple base interaction (G47:C16–G16) that is part of the PK helix (Figure 3A). These structural features are abbreviated here as P1x, PK and T, respectively.

To perturb the closed conformation, we designed four different mutants, named according to the location of mutation: P1x_C26Z, P1x_A14P, PK_C18A/G49U/C50U, and T_G16P. For P1x mutants, the original base pairing was altered by introducing a modified nucleotide: zebularine (Z: cytosine with N4 removed) or purine (P: adenine with N6 removed) (Figure 3A). For mutation of the PK helix, two original CG base pairings were replaced with weaker pairings: AU and GU. For mutation of the base triple T_G16P, G16 was replaced by purine, disrupting the interaction with the Hoogsteen edge of G47. In choosing the mutation sites, we avoided altering nucleotides that interact directly with the ligand to minimize disruption of the binding site. Additionally, the number of hydrogen bonds removed was kept to a minimum. The positions of these sequence variations can be found in Supplementary Figure S3, and the sequences of the mutants are listed in Supplementary Table S1.

All mutants exhibited an increase in the high FRET population with increasing ligand concentrations, showing that the mutations did not eliminate the ligand's ability to stimulate riboswitch folding (Supplementary Figure S4A–D). The fraction of the high FRET state versus ligand concentration could be fitted using a two-state binding isotherm, yielding *K*_d_ values (Table 1). Mutants that affect the PK helix stability (PK and T mutants) greatly reduced binding affinity: *K*_d_ (PK_C18A/G49U/C50U) > 1 mM; *K*_d_ (T_G16P) = 607 μM. In contrast, mutations at the P1x region had milder effects: *K*_d_ (P1x_C26Z: 41 μM; P1x_A14P: 61 μM).

**Table 1.** Binding affinity of SAM

Mutation	Binding affinity (*K*_d_) in μM
Wild-typed	11
P1x_C26Z	42
P1x_A14P	61
T_G16P	607
PK_C18A, G49U, C50U	>1000
P1xA14C, C26U	593

Next, we examined the impact of mutations on ligand-free folding dynamics and ligand responsiveness. In the absence of ligand, all mutants displayed peaks at similar FRET values as the wild type, indicating that open and closed conformations themselves were not significantly altered, but their relative populations changed (Figure 3B and C). For example, the destabilization of the closed conformation in the PK_C18A/G49U/C50U mutant led to a near-complete depletion of the closed conformations (Figure 3D middle panel). The other three mutants showed a more modest decrease in high FRET peak, by <3% for P1x_C26Z, 20% for P1x_A14P and 22% for T_G16P (Figure 3B and C). Considering both the binding affinity and the ligand-free conformations, our findings provide evidence for the notion that greater disruptions to the original ligand-free conformations result in greater reduction in binding affinity.

Upon ligand introduction, the general behavior observed in the wild-type riboswitch was observed for all mutants, but the relative populations and their ligand-induced changes were mutation-dependent (Figure 3D and Supplementary Figure S5). Compared to mutants targeting the extended P1 stem, mutants that were specifically designed to reduce the PK helix stability (PK_C18A/G49U/C50U and T_G16P) exhibited larger changes in conformation, corresponding to reduced binding affinities. As an example, the PK_C18A/G49U/C50U mutant had the constant high FRET population almost depleted, replaced by the dominant constant mid FRET populations (Figure 3E). Additionally, in the presence of the ligand, the dynamic population became more prevalent at the expense of the constant mid FRET population, while the constant high FRET population still remained nearly depleted (Figure 3E).

We also tested another mutant P1x_A14C/C26U, which stabilizes the extended P1 helix by introducing extra hydrogen bond (P1x_A14C, C26U) by replacing two original non-WC base pairs (*cis* sugar-Hoogsteen A13:C26, trans Hoogsteen-sugar A14:G25) with WC base pairs (A13:U26, C14:G25). Despite the introduction of extra hydrogen bonds, the mutants showed a reduction in closed conformation (Figure 3C bottom panel). In addition, the dynamic species increased in population accompanied by a loss of the constant high FRET species (Figure 3D). The substitution of the WC base pairs may have disturbed the original stacking geometry of the three extended P1 base pairs, resulting in less stable PK conformation and affecting the base pair C15:G24 that interacts with the ligand, and thus leading to a reduced binding affinity (*K*_d_ = 593 μM). These findings highlight the complex nature of riboswitch folding and how small, localized changes can alter the overall folding equilibrium and responsiveness to ligands, ultimately impacting binding affinity.

### Ribosome accessibility assay in the absence of ligand

**Figure 4.**
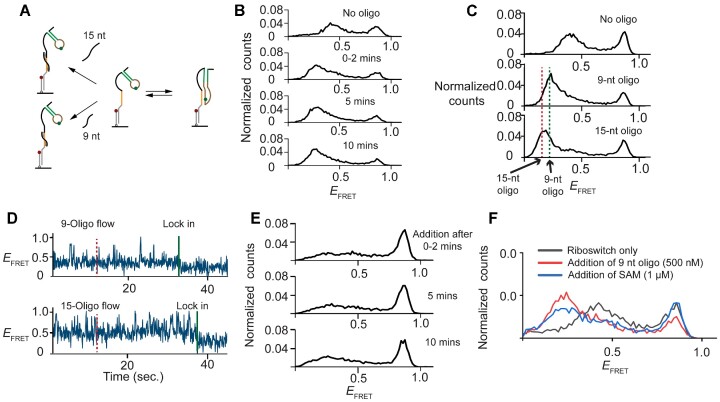
Pseudo-functional studies for assessing the accessibility of the translation initiation site. (**A**) A scheme showing 9 or 15-nt complementary oligonucleotides bind to the open conformation of the SAM/SAH riboswitch. (**B**) Distribution of *E*_FRET_ before and after addition of saturated concentration (= 500 nM) with various time points. (**C**) Distribution of *E*_FRET_ comparing the oligo-free (top), 9-nt (middle) and 15-nt (bottom) oligonucleotide-bound conformations. (**D**) Typical trajectories of riboswitches showed population converted from the mid-FRET to low FRET while oligonucleotides were flowed into the reaction chamber at 12 s, corresponding to the generated conformations observed in distribution of *E*_FRET_. (**E**) Distribution of *E*_FRET_ after simultaneous addition of 9-nt oligonucleotides (= 500 nM) and SAM (= 1 μM) at various time points, both additions are at saturated concentration. (**F**) Distribution of *E*_FRET_ showing the ligand responsiveness after the riboswitches were pre-bound by oligonucleotides.

We next explored the riboswitch function of blocking translational initiation in a ligand dependent manner by mimicking ribosome binding. The 3′ region of the riboswitch contains Shine–Dalgarno (S–D) site to which the ribosome binds to initiate translation. To fully cover the S–D sequence and form stable binding, we used a 9 nt oligonucleotide complementary to the S–D region in assessing the accessibility of the translation initiation site (Figure 4A). Binding of this oligonucleotide was easily monitored using our single molecule experiment.

In the absence of ligands, adding the oligonucleotides decreased the two major FRET populations (*E*_FRET_ = 0.4 & 0.84) and created a new population with an *E*_FRET_ of approximately 0.22 (Figure 4B). To identify a condition that the oligonucleotides are saturated to the pre-exposed S–D region, we conducted experiments with varying concentrations of oligonucleotides, and no discernible changes in conformation were observed above 500 nM (Supplementary Figure S6A). Consequently, all subsequent experiments were performed with the saturating oligonucleotide concentration of 500 nM.

We attribute the low-FRET state to the lengthening of 9 nt S–D region upon riboswitch-oligonucleotide complex formation. A similar experiment using a 15 nt oligonucleotide showed the low-FRET peak at a slightly lower value (∼0.18), consistent with the longer helix that would be formed (Figure 4C and Supplementary Figure S6B). Duplex formation was very stable and persisted even after washing away free oligonucleotides (Supplementary Figure S6C), in contrast to transient duplex formation observed using a shorter 7 nt long ribosome mimic for 7-aminomethyl-7-deazaguanine (preQ_1_)-sensing riboswitch (47).

We further examined the oligonucleotide binding reaction in real time by flowing in the oligonucleotide during observation. Most molecules remained unchanged or showed photobleaching because oligo binding generally took longer than our observation window of ∼180 s. Among molecules showing evidence of oligonucleotide binding, dynamic fluctuations between 0.4 and 0.84 FRET states were observed before they were locked into the low-FRET state (Figure 4D). In addition, most low-FRET states (75% and 91% for the 9-nt oligo and 15-nt oligo, respectively) were reached from the mid-FRET, open conformation (Figure 4D), indicating that the S–D region becomes accessible in the open conformation prior to oligonucleotide binding.

### Ligand-induced conformational change outcompetes ribosome mimic binding

We next examined the effect of the SAM ligand on the S–D region accessibility and riboswitch folding by simultaneously adding the ligand SAM and oligonucleotide ribosome mimics. Within min, the high-FRET population increased at the expense of the mid-FRET population. Only a small fraction of molecules (< 20%) went to the low-FRET conformation corresponding to the ribosome-mimic bound state (Figure 4E). Therefore, under our experimental conditions (1 mM SAM and 500 nM oligonucleotide), ligand binding outcompetes 9-nt oligonucleotide binding at early timepoints.

The riboswitch remained in the high-FRET state for the whole observation window for the 9-nt oligonucleotide, up to 60 min. However, for the 15-nt oligonucleotides, ∼40% of high-FRET conformation was lost by 10 min and by 1 h, the low-FRET conformation became dominant (Supplementary Figure S6D), suggesting that the ligand bound riboswitch can still undergo occasional visits to the open conformation. The additional foothold of the 15-nt oligo enables capturing the transiently exposed S–D site. At least for the longer ribosome-mimic, the equilibrium favors the oligo-bound ‘gene-on’ state whereas early in the process, ligand binding can trap the molecule in the closed conformation, momentarily blocking access to the ribosome. However, it is also possible that the extra foothold for the long oligo may facilitate pseudoknot unwinding by partially hybridizing to an unpaired region followed by strand displacement.

To test if the oligo-bound riboswitch remains responsive to ligand, we added the ligand after pre-incubation with oligonucleotide. Only ∼25% of 9-nt oligo-bound structures converted to the ligand-bound (*E*_FRET_ = 0.84 state), and a negligible fraction of the 15-nt oligo-bound riboswitch was responsive to the ligand (Figure 4F and Supplementary Figure S6E). Therefore, to function as a translational riboswitch, the decision must be made before the arrival of the ribosome, assuming ribosome binding happens only once. In the more realistic case of multiple ribosome molecules arriving and initiating translation in succession, the riboswitch activity can be gradational.

### Vectorial folding assay disfavors ligand binding compared to the pre-folded riboswitch

**Figure 5.**
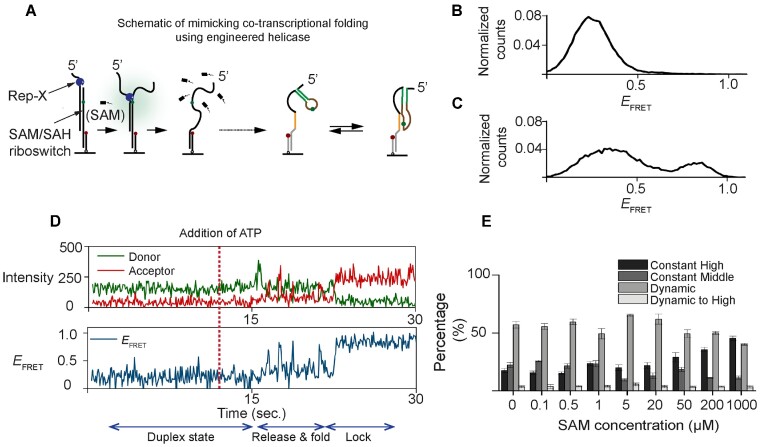
Vectorially folded assays for mimicking co-transcriptional folding. (**A**) A scheme showing the engineered superhelicase Rep-X was preincubated and initiated at designated time for unwinding the heteroduplex. The riboswitch was hybridized with a complementary DNA oligonucleotide to form an RNA-DNA heteroduplex with a 3′ overhang at the DNA terminus. The same Cy3–Cy5 FRET pair was used to determine the riboswitch conformations. (**B**) Distribution of *E*_FRET_ before introducing ATP, corresponding to the heteroduplex conformation. (**C**) Distribution of *E*_FRET_ after vectorially folding, corresponding to the conformations released from the heteroduplex. (**D**) A typical trajectory showing a heteroduplex is unwound and folded into the constant high FRET conformation, where the ATP is flowed in at 12 s. (**E**) Histograms of relative populations in the presence of various SAM concentrations.

Because RNA folds co-transcriptionally, riboswitch function should be examined in the context of ongoing transcription (48). Several different methods of mimicking co-transcriptional riboswitch folding and function are available (44,45,49–55). Here we used the vectorial folding assay where a DNA helicase is used to mimic co-transcriptional RNA folding (44,45). The riboswitch was hybridized with a complementary DNA oligonucleotide to form an RNA-DNA heteroduplex with a 3′ overhang at the DNA terminus. The same Cy3–Cy5 FRET pair as in our previous experiments was used to determine the riboswitch conformation (Figure 5A). A highly processive, engineered DNA helicase, Rep-X (46), was used to unwind the heteroduplex unidirectionally by translocating on the DNA strand in the 3′ to 5′ direction, to release the RNA strand progressively in the 5′ to 3′ direction of transcription and at the speed of transcription, about ∼60 nt per second (44,45).

Upon Rep-X addition without ATP, we observed low FRET efficiency (*E*_FRET_ = 0.2) because the fluorophores remain separated by the heteroduplex (Figure 5B). After ATP addition, two new populations centered at 0.4 and 0.84 emerged, corresponding to the open and closed conformations, respectively (Figure 5C). A representative vectorial folding trace shows two features (Figure 5D). First, Cy3 intensity shows a transient increase due to protein-induced fluorescence enhancement (56,57), signifying Rep-X approaching the Cy3 fluorophore on the RNA strand. Second, the heteroduplex unwinds and riboswitch folding begins. We classified the riboswitch folding behavior into four types (Supplementary Figure S7A) : (i) molecules that transitioned from the heteroduplex state to the stable closed conformation without any detectable intermediate, (ii) molecules that transitioned from the heteroduplex state to the stable open conformation, (iii) molecules that transitioned from the heteroduplex state to one undergoing fluctuations between the open and closed conformations and (iv) molecules that transitioned from the heteroduplex to the stable closed conformation after first fluctuating between open and closed states. These distinct populations are in agreement with the observed conformations for pre-folded riboswitches except for the type (iv), likely because this behavior is observable only on the path to reach folding equilibrium.

Addition of ligand during vectorial folding changed the relative populations of the four types of folding behavior. Type I, direct transition to stable high-FRET state, became more populated at higher ligand concentrations (Figure 5E) and its fraction vs ligand concentration could be well fitted using a two-state binding isotherm (Supplementary Figure S7B), yielding an apparent *K*_d_ value of 108 μM. This apparent *K*_d_ is an order of magnitude higher than the *K*_d_ value of 10 μM we observed for pre-folded RNA, suggesting ligand-responsive conformation is not immediately obtained during co-transcriptional folding. We hypothesized that the increase in *K*_d_ is due to insufficient time for the nascent riboswitch to reach the steady-state conformations. Indeed, as shown in Supplementary Figure S7C, the conformational analyses of time points after vectorial folding exhibited noticeable differences. Specifically, the conformations observed at 5 seconds post-folding displayed a lower fraction of high-FRET population and decreased responsiveness to ligand. These results imply that the nascent riboswitch necessitates more than a few seconds to attain a steady state conformational distribution, thereby reducing its ligand-binding affinity during transcription.

### Vectorial folding favors ribosome mimic binding

**Figure 6.**
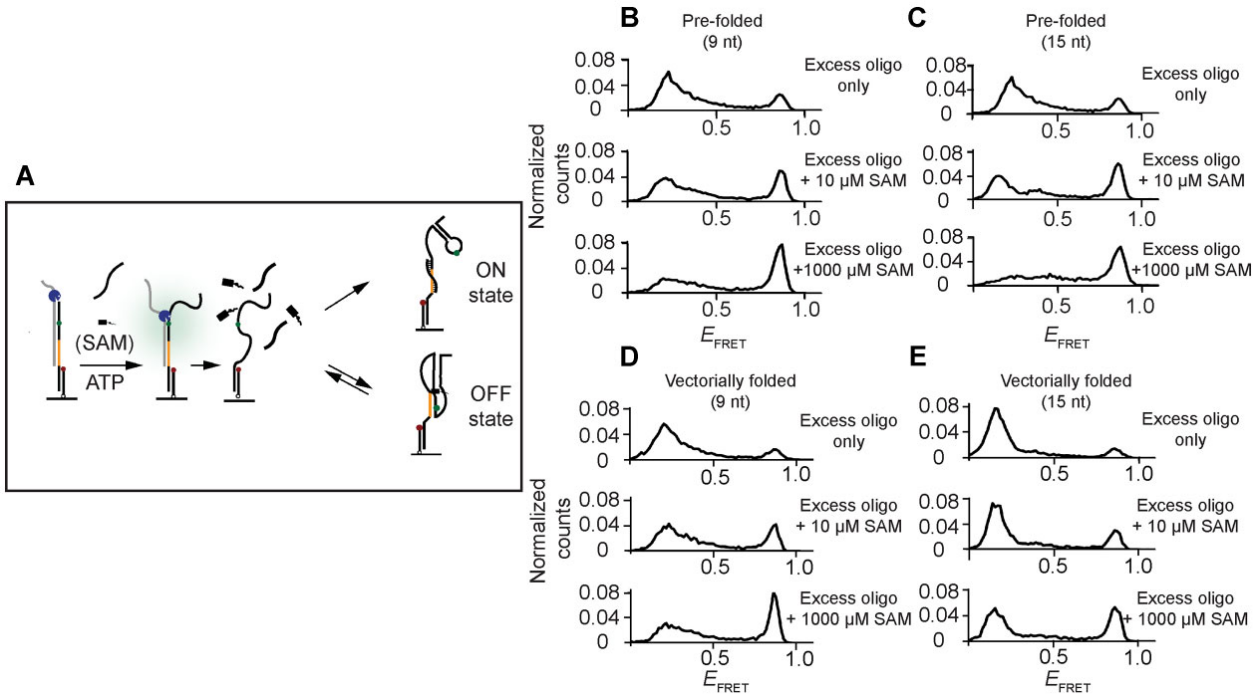
Pseudo-functional studies for assessing the accessibility of the translation initiation site during vectorially folding. (**A**) A scheme showing SAM, oligonucleotides, and ATP are flowed in simultaneously for simulating competition over mutually exclusive conformations during co-transcriptional folding. The oligonucleotide-bound state is termed ‘ON’ state, indicating translation can be initiated, whereas the ligand-bound state is termed ‘OFF’ state, indicating the Shine–Dalgarno site is blocked. (**B**) Distribution of *E*_FRET_ for pre-folded riboswitches under simultaneous addition of 9-nt oligonucleotides and various concentrations of SAM. Similar competitions between oligonucleotides and ligands were carried out while the riboswitches are vectorially folded and distribution of *E*_FRET_ with various SAM concentrations is shown in (**D**). Similar competition experiments were carried out with longer 15-nt oligonucleotides, for pre-folded competition, (**C**) distribution of *E*_FRET_ with various SAM concentrations; for vectorially folded competition (**E**) distribution of *E*_FRET_ with various SAM concentrations.

In the ribosome accessibility assay on pre-folded riboswitches, we found that ligand binding is kinetically favored over ribosome mimic binding. To test if this result holds for vectorial folding, we included saturating concentration of-9 nt or 15-nt oligonucleotides during vectorial folding (Figure 6A). In the absence of ligand, the closed conformation was rarely observed, likely because the S–D region revealed through heteroduplex unwinding is bound by the ribosome mimic before the aptamer can fold into the closed form (top histogram of Figure 6D and E). This finding is also in line with our observation that it takes min for the nascent riboswitch to attain a steady-state conformational distribution (as demonstrated in Supplementary Figure S7C).

In the presence of ligand, the closed conformation was obtained in a ligand-concentration dependent manner. The efficacy of the ligand in converting the riboswitch to its closed conformation state was diminished in the vectorial folding condition compared to the pre-folded condition for both ribosome mimics (Figure 6B–E and Supplementary Figure S9). The nascent riboswitch likely first adopts the open conformation, which facilitates ribosome mimic binding, and reduces the mutually exclusive ligand bound conformation.

## DISCUSSION

We propose a model describing the folding scheme and its energy landscape based on our findings of multiple populations of static folds, open or closed and dynamic switching, and the highly heterogenous switching rates. The FRET values (0.4 and 0.84) of the switching molecules were indifferentiable from those with static conformations, suggesting there is significant structural resemblance. We were surprised that the majority (∼55%) of ligand-free riboswitches showed dynamic switching between the closed and open conformations. Most of the dynamically switching molecules are responsive to ligand, either by population conversion to the static closed conformation or rate alterations. The observed heterogeneity is likely to be a property inherent to the riboswitch because our constructs with their modifications for surface tethering and fluorescence imaging showed comparable binding affinity to what was determined from unmodified RNA in bulk solution. Furthermore, all five single-site mutants we tested show similarly heterogeneous behavior with only their relative populations and kinetics changed.

Mutants examined in this study showed that not only the populations of static and dynamic populations were strongly affected, but the rates of switching between conformations changed (Supplementary Figure S8). We speculate that any incomplete base pair formation of the extended P1 stem (E-P1) or PK helix may introduce metastable conformations, leading to heterogeneous folding/unfolding rates for this riboswitch, and potentially for other functional RNAs that also contain the H-type pseudoknot (58–61). Such heterogeneity, if present *in vivo*, may buffer the riboswitch activity against a wide range of ligand concentrations.

Our findings are most consistent with the previously proposed hybrid model combining conformation selection & induced-fit (10): whereas all conformations are sampled in the absence of ligand (conformation selection), ligand addition repopulates the population ensemble by imparting further stability to the ligand-bound state (induced-fit). A previous SAM-II riboswitch study reported that transient conformational excursions occur in the absence of ligand, suggesting conformational sampling (10). However, they could not determine if those transient conformations were responsive to ligands or how folding and ligand binding are promoted through specific structural motifs.

Relevant to our evaluation of the riboswitch's accessibility for ribosome mimics, a previous study probed the folding of the 7-aminomethyl-7-deazaguanine-sensing riboswitch using a 7 nt long fluorescently labeled oligonucleotides as translational initiation mimic (46). They observed bursts of probe binding and showed that ligand addition reduces burst duration and extends the intervals between bursts. However, the use of fluorescently labeled probes limited their analysis to sub-*K*_d_ concentrations. By employing unlabeled oligonucleotides, we were able to mimic translation initiation under conditions of saturating ribosome mimic so that the exposure of the binding site is rate-limiting and show that the nascent folds adopted have yet to reach an equilibrium, thus leading to a reduced ligand binding affinity.

In the vectorial folding assay, we observed a decrease in ligand binding affinity (Figure 6 and Supplementary Figure S9), resulting in a reduction in the effectiveness of ligand binding when competing with a ribosome mimic. These differences between pre-folded and vectorially folded riboswitches suggest that the timing of regulatory decision is critical to the effectiveness of the riboswitch and may explain the requirement for higher ligand concentration for effective regulatory control *in vivo* (62). It is possible that there are different modes of regulating accessibility, and the timing of transcription and translation coupling. For tighter regulation, riboswitch needs to reach equilibrium first, thus transcription needs to be carried out in advance of translation. However, when regulation needs not to be tight, the transcription and translation can happen simultaneously.

In conclusion, our studies on this small SAM/SAH riboswitch provide valuable insights into the complexities of the folding landscape, including individual folding heterogeneity and the role of RNA folding kinetics. Furthermore, our findings have implications for the translational control governed by the riboswitch, highlighting the critical influence of folding equilibrium on the efficacy of regulatory decisions.

## Supplementary Material

gkad633_Supplemental_File

## Data Availability

Analyses and data acquisition codes are upload on lab GitHub account and archived in Zenodo with the following doi. Additionally, raw data that support our findings have been uploaded and archived in Zenodo, corresponding to each individual figure. GitHub: https://github.com/Ha-SingleMoleculeLab Analyses, data acquisition codes, and raw data are archived in Zenodo: Raw data analysis DOI: 10.5281/zenodo.4925617 Data acquisition DOI: 10.5281/zenodo.4925630 Raw data DOI: 10.5281/zenodo.8088172
